# Supplementary material for: Expanding diversity of tick-borne phleboviruses (Phlebovirus mukawaense, Mudanjiang phlebovirus, Gomselga Virus, and Onega tick phlebovirus) in Russia
Source: PLoS One. 2026 Jun 2;21(6):e0349564. doi: 10.1371/journal.pone.0349564 (PMC13229322; doi:10.1371/journal.pone.0349564)
Supplement: S1 Table — (PDF) [file pone.0349564.s001.pdf]

S1 Table. Oligonucleotide primers used for targeted library enrichment on NGS.

MKWV segment	Oligonucleotide name	Oligonucleotide sequence	Length, nt
S	MS1F	TAAAGAGCATTGCGGAGGAG	900
	MS1R	TTAACTTGTCCCAGAAACTGAG	
	MS2F	CTTAGGGAGCACCAAGAGCA	900
	MS2R	AAGAAGAACTGCAAGATGGGA	
M	MM1F	CAAATACTCCTCCTGCTCTC	1188
	MM1R	TGCCCTTCACCTTTATTGCT	
	MM2F	GACACCACCTTCTGCAATCAC	1008
	MM2R	TAACTACTGACCTCTGCTTGCT	
	MM3F	CACAAGCAAGCAGAGGTCAG	1000
	MM3R	CCGTACACATGCAGTTTCTTCTC	
L	ML1F	TTCAACACTGCACTACCATCC	1000
	ML1R	GGCTAGTCTCAGCTCCTCCT	
	ML2F	AGTCCACTGTCCAATTTCCT	1000
	ML2R	ACAAACCCTTCCATCATCAC	
	ML3F	TATGTGATGATGGAAGGGTTTGTC	900
	ML3R	CTGGGATTCTAGTCTTATTCTTGG	
	ML4F	CCCAAGAATAAGACTAGAATCCC	1081
	ML4R	CTCAGACCCTTCAGAAACCA	
	ML5F	TTGAGCACTATGCCAAACAC	938
	ML5R	TGAACATGAGAAGCAAGAGG	
	ML6F	CTTGCTTCTCATGTTCAGATCAG	752
	ML6R	GCTCACAATATAGACAGGGAC	
	ML7F	GCGTCCCTGTCTATATTGTGAG	854
	ML7R	TCTAGCCGAGCATGTCATCC	
