# Supplementary material for: Expanding diversity of tick-borne phleboviruses (Phlebovirus mukawaense, Mudanjiang phlebovirus, Gomselga Virus, and Onega tick phlebovirus) in Russia
Source: PLoS One. 2026 Jun 2;21(6):e0349564. doi: 10.1371/journal.pone.0349564 (PMC13229322; doi:10.1371/journal.pone.0349564)
Supplement: S2 Table — (PDF) [file pone.0349564.s002.pdf]

S2 Table. Sequences using for analysis in this study.

No.	GenBank ID	Virus name	Isolate/strain	Taxonomy	Isolation source	Geographic location name	Collection data
1	PP476824	Mukawa virus	Tuva-46	*Phlebovirus mukawaense*	*Ixodes persulcatus*	Russia	2023
2	PP476821	Mukawa virus	Chita-16	*Phlebovirus mukawaense*	*Ixodes persulcatus*	Russia	2023
3	PP476823	Mukawa virus	Chita-29	*Phlebovirus mukawaense*	*Ixodes persulcatus*	Russia	2023
4	PP476822	Mukawa virus	Chita-17	*Phlebovirus mukawaense*	*Ixodes persulcatus*	Russia	2023
5	PV843499	Mukawa virus	Primorye-50	*Phlebovirus mukawaense*	*Ixodes persulcatus*	Russia	2024
6	PP476819	Mukawa virus	Primorye-49	*Phlebovirus mukawaense*	*Ixodes persulcatus*	Russia	2023
7	NC043510	Mukawa virus	MKW73	*Phlebovirus mukawaense*	*Ixodes persulcatus*	Japan	2013
8	ON408129	Mukawa virus	NE-DH3	*Phlebovirus mukawaense*	*Ixodes persulcatus*	China	2020
9	OM066888	Mukawa virus	346-6443	*Phlebovirus mukawaense*	*Haemaphysalis concinna*	China	2021
10	PP476820	Mukawa virus	Primorye-62	*Phlebovirus mukawaense*	*Ixodes persulcatus*	Russia	2023
11	ON408126	Mukawa virus	NE-ShL2	*Phlebovirus mukawaense*	*Dermacentor silvarum*	China	2021
12	OR730564	Mukawa virus	GH1	*Phlebovirus mukawaense*	*Ixodes persulcatus*	China	2023
13	PP476827	Gomselga virus	Khakassia-28	*Phlebovirus*	*Ixodes persulcatus*	Russia	2023
14	PP476828	Gomselga virus	Irkutsk-4	*Phlebovirus*	*Ixodes persulcatus*	Russia	2023
15	PP476838	Gomselga virus	Tuva-39	*Phlebovirus*	*Ixodes persulcatus*	Russia	2023
16	PP476835	Gomselga virus	Tuva-7	*Phlebovirus*	*Ixodes persulcatus*	Russia	2023
17	PP476833	Gomselga virus	Irkutsk-55	*Phlebovirus*	*Ixodes persulcatus*	Russia	2023
18	PP476830	Gomselga virus	Irkutsk-34	*Phlebovirus*	*Ixodes persulcatus*	Russia	2023
19	PP476832	Gomselga virus	Irkutsk-53	*Phlebovirus*	*Ixodes persulcatus*	Russia	2023
20	PP476831	Gomselga virus	Irkutsk-35	*Phlebovirus*	*Ixodes persulcatus*	Russia	2023
21	PP476834	Gomselga virus	Tuva-3	*Phlebovirus*	*Ixodes persulcatus*	Russia	2023
22	PP476840	Gomselga virus	Tuva-45	*Phlebovirus*	*Ixodes persulcatus*	Russia	2023
23	PP476837	Gomselga virus	Tuva-31	*Phlebovirus*	*Ixodes persulcatus*	Russia	2023

24	PP476836	Gomselga virus	Tuva-26	*Phlebovirus*	*Ixodes persulcatus*	Russia	2023
25	PP476839	Gomselga virus	Tuva-40	*Phlebovirus*	*Ixodes persulcatus*	Russia	2023
26	MT380752	Gomselga virus	T27077	*Phlebovirus*	*Ixodes persulcatus*	Russia	2018
27	MT380757	Gomselga virus	T28815	*Phlebovirus*	*Ixodes persulcatus*	Russia	2018
28	PP476829	Gomselga virus	Irkutsk-29	*Phlebovirus*	*Ixodes persulcatus*	Russia	2023
29	PP476826	Gomselga virus	Khakassia-21	*Phlebovirus*	*Ixodes persulcatus*	Russia	2023
30	PP476825	Gomselga virus	Khakassia-10	*Phlebovirus*	*Ixodes persulcatus*	Russia	2023
31	ON920443	Gomselga virus	GOM-Che-T22645	*Phlebovirus*	*Ixodes persulcatus*	Russia	2014
32	MT380754	Gomselga virus	T27359	*Phlebovirus*	*Ixodes persulcatus*	Russia	2018
33	MT380749	Gomselga virus	T16420	*Phlebovirus*	*Ixodes persulcatus*	Russia	2012
34	PP476844	Mudanjiang phlebovirus	Primorye-29	*Phlebovirus*	*Ixodes persulcatus*	Russia	2023
35	OR148743	Mudanjiang phlebovirus	MDJV_BSQG1731	*Phlebovirus*	*Ixodes persulcatus*	China	2017
36	ON408132	Mudanjiang phlebovirus	NE-FZ3	*Phlebovirus*	*Ixodes persulcatus*	China	2021
37	OR723773	Mudanjiang phlebovirus	HLJ-26	*Phlebovirus*	*Ixodes persulcatus*	China	2021
38	JF838324	Chize virus	Brest Ar/T2913	*Uukuvirus uukuniemiense*	*Ixodes (Trichotoixodes) frontalis*	France	1993
39	KJ425423	Gissar virus	5595	*Uukuvirus uukuniemiense*	*Argas reflexus* (tick)	Tajikistan	1982
40	PP476841	Onega tick phlebovirus	Irkutsk-18	*Phlebovirus*	*Ixodes persulcatus*	Russia	2023
41	PP476842	Onega tick phlebovirus	Irkutsk-23	*Phlebovirus*	*Ixodes persulcatus*	Russia	2023
42	PP476843	Onega tick phlebovirus	Irkutsk-33	*Phlebovirus*	*Ixodes persulcatus*	Russia	2023

43	MN542366	Onega tick phlebovirus	Rus/Ix_persulcatus/Karelia/3/2018	*Phlebovirus*	*Ixodes persulcatus*	Russia	2018
44	OQ185282	Onega tick phlebovirus	GH1	*Phlebovirus*	*Ixodes persulcatus*	China	2022
45	OR148781	Onega tick phlebovirus	OTPV_THQG1704	*Phlebovirus*	*Ixodes persulcatus*	China	2017
46	ON408150	Onega tick phlebovirus	NE-TH3	*Phlebovirus*	*Ixodes persulcatus*	China	2021
47	NC055432	Blacklegged tick phlebovirus 3	A1		*Ixodes scapularis*	USA	2014
48	MW199200	Elliovirales sp.	L1b		Ticks	China	2021
49	PP476845	Phenuiviridae sp.	Tuva-21		*Ixodes persulcatus*	Russia	2023
50	HM802202	Phlebovirus SD4/China/2010	SD4	*Bandavirus dabieense*	*Homo sapiens*	China	2010
51	JN258707	Phlebovirus JN1/China/2010	JN1/China/2010	*Bandavirus dabieense*	*Homo sapiens*	China	2010
52	NC078350	Bogoria virus	SP105-KE-2016	*Phlebovirus bogoriaense*	*Phlebotominae*	Kenya	2016
53	NC055319	Gabek Forest virus	Sud AN 754-61	*Phlebovirus gabekense*	*Acomys cahirinus*	Sudan	1961
54	NC078061	Saint Floris virus	Dak ANB 512	*Phlebovirus florisense*	*Tatera sp.*	Central African Republic	1971
55	JF939846	Tehran virus	I-47	*Phlebovirus tehranense*	*Phlebotomus perfiliewi*	Iran	1976
56	OP554808	Toscana virus	TOSV/Spain/ LCR_853/2019	*Phlebovirus toscanaense*	*Homo sapiens*	Spain	2019
57	PP525079	Mukawa virus	Primorye-49	*Phlebovirus mukawaense*	*Ixodes persulcatus*	Russia	2023
58	PP525081	Mukawa virus	Primorye-62	*Phlebovirus mukawaense*	*Ixodes persulcatus*	Russia	2023
59	OR730570	Mukawa virus	GH1	*Phlebovirus mukawaense*	*Ixodes persulcatus*	China	2023
60	ON408119	Mukawa virus	NE-YC4	*Phlebovirus mukawaense*	*Ixodes persulcatus*	China	2021

61	PP525078	Mukawa virus	Chita-29	*Phlebovirus mukawaense*	*Ixodes persulcatus*	Russia	2023
62	PP525076	Mukawa virus	Chita-16	*Phlebovirus mukawaense*	*Ixodes persulcatus*	Russia	2023
63	PP525077	Mukawa virus	Chita-17	*Phlebovirus mukawaense*	*Ixodes persulcatus*	Russia	2023
64	PQ215523	Mukawa virus	HLJ1	*Phlebovirus mukawaense*	*Ixodes persulcatus*	China	2023
65	PP999502	Mukawa virus	NAC-Org5	*Phlebovirus mukawaense*	*Homo sapiens*	China	2023
66	ON408125	Mukawa virus	NE-FZ3	*Phlebovirus mukawaense*	*Ixodes persulcatus*	China	2021
67	ON408122	Mukawa virus	NE-FZ2	*Phlebovirus mukawaense*	*Ixodes persulcatus*	China	2021
68	ON408131	Mukawa virus	NE-DH3	*Phlebovirus mukawaense*	*Ixodes persulcatus*	China	2020
69	PP525080	Mukawa virus	Primorye-50	*Phlebovirus mukawaense*	*Ixodes persulcatus*	Russia	2023
70	PP525075	Mukawa virus	Tuva-46	*Phlebovirus mukawaense*	*Ixodes persulcatus*	Russia	2023
71	PP473636	Mukawa virus	HL4	*Phlebovirus mukawaense*	*Ixodes persulcatus*	China	2022
72	NC043511	Mukawa virus	MKW73	*Phlebovirus mukawaense*	*Ixodes persulcatus*	Japan	2013
73	OM066890	Mukawa virus	346-6443	*Phlebovirus mukawaense*	*Haemaphysalis concinna*	China	2021
74	MZ532500	Mukawa virus		*Phlebovirus mukawaense*	*Ixodes persulcatus*	China	2020
75	OR730568	Mukawa virus	YKS1	*Phlebovirus mukawaense*	*Ixodes persulcatus*	China	2023
76	ON408128	Mukawa virus	NE-ShL2	*Phlebovirus mukawaense*	*Dermacentor silvarum*	China	2021
77	PP473637	Mukawa virus	HL6	*Phlebovirus mukawaense*	*Ixodes persulcatus*	China	2022
78	ON408116	Mukawa virus	NE-TH3	*Phlebovirus mukawaense*	*Ixodes persulcatus*	China	2021
79	OR730569	Mukawa virus	XQ1	*Phlebovirus mukawaense*	*Ixodes persulcatus*	China	2023
80	OP863272	Mukawa virus	NM-DS-8	*Phlebovirus mukawaense*	*Dermacentor silvarum*	China	2020
81	PQ215522	Mukawa virus	HLJ1	*Phlebovirus mukawaense*	*Ixodes persulcatus*	China	2023
82	PP999501	Mukawa virus	NAC-Org5	*Phlebovirus mukawaense*	*Homo sapiens*	China	2023
83	ON408124	Mukawa virus	NE-FZ3	*Phlebovirus mukawaense*	*Ixodes persulcatus*	China	2021
84	ON408121	Mukawa virus	NE-FZ2	*Phlebovirus mukawaense*	*Ixodes persulcatus*	China	2021
85	ON408130	Mukawa virus	NE-DH3	*Phlebovirus mukawaense*	*Ixodes persulcatus*	China	2020

86	MZ532499	Mukawa virus		*Phlebovirus mukawaense*	*Ixodes persulcatus*	China	2020
87	ON408127	Mukawa virus	NE-ShL2	*Phlebovirus mukawaense*	*Dermacentor silvarum*	China	2021
88	PP473634	Mukawa virus	HL4	*Phlebovirus mukawaense*	*Ixodes persulcatus*	China	2022
89	PV093713	Mukawa virus	NM2YC/2021	*Phlebovirus mukawaense*	*Ixodes persulcatus*	China	2021
90	PV093710	Mukawa virus	NM3YT/2021	*Phlebovirus mukawaense*	*Ixodes persulcatus*	China	2021
91	OR730567	Mukawa virus	GH1	*Phlebovirus mukawaense*	*Ixodes persulcatus*	China	2023
92	OM066889	Mukawa virus	346-6443	*Phlebovirus mukawaense*	*Haemaphysalis concinna*	China	2021
93	NC043509	Mukawa virus	MKW73	*Phlebovirus mukawaense*	*Ixodes persulcatus*	Japan	2013
94	PP473635	Mukawa virus	HL6	*Phlebovirus mukawaense*	*Ixodes persulcatus*	China	2022
95	OR730566	Mukawa virus	XQ1	*Phlebovirus mukawaense*	*Ixodes persulcatus*	China	2023
96	OR730565	Mukawa virus	YKS1	*Phlebovirus mukawaense*	*Ixodes persulcatus*	China	2023
97	PP525068	Mukawa virus	Tuva-46	*Phlebovirus mukawaense*	*Ixodes persulcatus*	Russia	2023
98	ON408115	Mukawa virus	NE-TH3	*Phlebovirus mukawaense*	*Ixodes persulcatus*	China	2021
99	PP525069	Mukawa virus	Chita-16	*Phlebovirus mukawaense*	*Ixodes persulcatus*	Russia	2023
100	PP525070	Mukawa virus	Chita-17	*Phlebovirus mukawaense*	*Ixodes persulcatus*	Russia	2023
101	PP525071	Mukawa virus	Chita-29	*Phlebovirus mukawaense*	*Ixodes persulcatus*	Russia	2023
102	ON408118	Mukawa virus	NE-YC4	*Phlebovirus mukawaense*	*Ixodes persulcatus*	China	2021
103	PP525072	Mukawa virus	Primorye-49	*Phlebovirus mukawaense*	*Ixodes persulcatus*	Russia	2023
104	PP525073	Mukawa virus	Primorye-50	*Phlebovirus mukawaense*	*Ixodes persulcatus*	Russia	2023
105	PP525074	Mukawa virus	Primorye-62	*Phlebovirus mukawaense*	*Ixodes persulcatus*	Russia	2023
106	PQ215521	Mukawa virus	HLJ1	*Phlebovirus mukawaense*	*Ixodes persulcatus*	China	2023
107	PP999500	Mukawa virus	NAC-Org5	*Phlebovirus mukawaense*	*Homo sapiens*	China	2023
108	ON408120	Mukawa virus	NE-FZ2	*Phlebovirus mukawaense*	*Ixodes persulcatus*	China	2021
109	ON408123	Mukawa virus	NE-FZ3	*Phlebovirus mukawaense*	*Ixodes persulcatus*	China	2021
110	ON408126	Mukawa virus	NE-ShL2	*Phlebovirus mukawaense*	*Dermacentor silvarum*	China	2021

111	ON812249	Mukawa virus	TIGMIC 2	*Phlebovirus mukawaense*	*Ixodes persulcatus*	China	2019
112	ON812167	Mukawa virus	TIGMIC 1	*Phlebovirus mukawaense*	*Ixodes persulcatus*	China	2019
113	PV093718	Mukawa virus	NM3YL/ 2021 scaffold_ 6426	*Phlebovirus mukawaense*	*Ixodes persulcatus*	China	2021
114	PV093715	Mukawa virus	NM3YL/ 2021 scaffold_ 6413	*Phlebovirus mukawaense*	*Ixodes persulcatus*	China	2021
115	PP525066	Mukawa virus	Primorye -50	*Phlebovirus mukawaense*	*Ixodes persulcatus*	Russia	2023
116	PP525067	Mukawa virus	Primorye -62	*Phlebovirus mukawaense*	*Ixodes persulcatus*	Russia	2023
117	ON812263	Mukawa virus	TIGMIC 4	*Phlebovirus mukawaense*	*Ixodes persulcatus*	China	2019
118	OR730564	Mukawa virus	GH1	*Phlebovirus mukawaense*	*Ixodes persulcatus*	China	2023
119	ON408117	Mukawa virus	NE-YC4	*Phlebovirus mukawaense*	*Ixodes persulcatus*	China	2021
120	PV093712	Mukawa virus	NM2YC/ 2021	*Phlebovirus mukawaense*	*Ixodes persulcatus*	China	2021
121	PV093709	Mukawa virus	NM3YT/ 2021	*Phlebovirus mukawaense*	*Ixodes persulcatus*	China	2021
122	PP473632	Mukawa virus	HL4	*Phlebovirus mukawaense*	*Ixodes persulcatus*	China	2022
123	OM066888	Mukawa virus	346-6443	*Phlebovirus mukawaense*	*Haemaphysalis concinna*	China	2021
124	ON408114	Mukawa virus	NE-TH3	*Phlebovirus mukawaense*	*Ixodes persulcatus*	China	2021
125	PP473633	Mukawa virus	HL6	*Phlebovirus mukawaense*	*Ixodes persulcatus*	China	2022
126	OR730562	Mukawa virus	YKS1	*Phlebovirus mukawaense*	*Ixodes persulcatus*	China	2023
127	PP525061	Mukawa virus	Tuva-46	*Phlebovirus mukawaense*	*Ixodes persulcatus*	Russia	2023
128	OR730563	Mukawa virus	XQ1	*Phlebovirus mukawaense*	*Ixodes persulcatus*	China	2023
129	PP525062	Mukawa virus	Chita-16	*Phlebovirus mukawaense*	*Ixodes persulcatus*	Russia	2023
130	PP525063	Mukawa virus	Chita-17	*Phlebovirus mukawaense*	*Ixodes persulcatus*	Russia	2023
131	PP525064	Mukawa virus	Chita-29	*Phlebovirus mukawaense*	*Ixodes persulcatus*	Russia	2023
132	ON408129	Mukawa virus	NE-DH3	*Phlebovirus mukawaense*	*Ixodes persulcatus*	China	2020
133	ON812314	Mukawa virus	TIGMIC 8	*Phlebovirus mukawaense*	*Ixodes persulcatus*	China	2019

134	ON812303	Mukawa virus	TIGMIC 6	*Phlebovirus mukawaense*	*Ixodes persulcatus*	China	2019
135	PP525065	Mukawa virus	Primorye -49	*Phlebovirus mukawaense*	*Ixodes persulcatus*	Russia	2023
136	ON812312	Mukawa virus	TIGMIC 7	*Phlebovirus mukawaense*	*Ixodes persulcatus*	China	2019
137	NC043510	Mukawa virus	MKW73	*Phlebovirus mukawaense*	*Ixodes persulcatus*	Japan	2013
138	OP863271	Mukawa virus	NM-DS-7	*Phlebovirus mukawaense*	*Dermacentor silvarum*	China	2020
