# Supplementary material for: Expanding diversity of tick-borne phleboviruses (Phlebovirus mukawaense, Mudanjiang phlebovirus, Gomselga Virus, and Onega tick phlebovirus) in Russia
Source: PLoS One. 2026 Jun 2;21(6):e0349564. doi: 10.1371/journal.pone.0349564 (PMC13229322; doi:10.1371/journal.pone.0349564)
Supplement: S3 Table — (PDF) [file pone.0349564.s003.pdf]

S3 Table. Comparison of MKWV putative protein tertiary structures with those of homologous proteins from closely related viruses.

PDB ID	Description	Taxonomy	Sequence identity	TM-score
**S segment**				
**MKWV Nucleoprotein, 247 a.a.**				
4csf-assembly1_X	Structural insights into Toscana virus RNA encapsidation	*Toscana virus*	41.3	0.908
4h5o-assembly1_B	Crystal Structure of Rift Valley Fever Virus Nucleocapsid Protein	*Rift Valley fever virus*	45.9	0.901
4j4w assembly1_A	Crystal structure of BueVN	*Buenaventura virus*	44.9	0.900
**MKWV NS protein, 340 a.a.**				
7rlo assembly1_L	Structure of the human eukaryotic translation initiation factor 2B	Sandfly fever Sicilian virus	17.2	0.672
7f67 assembly1_L	eIF2B-SFSV NSs-2-eIF2	Sandfly fever Sicilian virus	18.6	0.666
7f66 assembly1_K	eIF2B-SFSV NSs-1-eIF2	Sandfly fever Sicilian virus	19.1	0.663
**M segment**				
**MKWV glycoprotein G1, 522 a.a.**				
5y0y assembly1_A	RVFV GN-AU	*Rift Valley fever virus*	21.3	0.638
6iea assembly1_A	Structure of RVFV Gn and human monoclonal antibody R13	*Rift Valley fever virus*	20.7	0.638
6iek assembly2_D	Structure of RVFV Gn and human monoclonal antibody R12	*Rift Valley fever virus*	20.1	0.638
6iec assembly4_I	Structure of RVFV Gn and human monoclonal antibody R17	*Rift Valley fever virus*	20.5	0.638
**MKWV glycoprotein G2 fusion domain, 312 a.a.**				
6egt assembly1_B	Structure of RVFV envelope protein Gc in postfusion conformation in complex with MES	*Rift Valley fever virus*	38.4	0.722
5g47 assembly1_C	Structure of Gc glycoprotein from severe fever with thrombocytopenia syndrome virus in trimeric postfusion conformation	*Severe fever with thrombocytopenia syndrome virus*	24.1	0.709
6egu assembly1_B	Structure of RVFV envelope protein Gc in postfusion conformation in complex with 1,2-diproionyl-sn-glycero-3-phosphocholine	*Rift Valley fever virus*	39	0.729
6egu assembly1_C	Structure of RVFV envelope protein Gc in postfusion	*Rift Valley fever virus*	38.5	0.725

	conformation in complex with 1,2-diproionyl-sn-glycero-3-phosphocholine			
**MKWV glycoprotein G2 C-terminal domain, 86 a.a.**				
1z8y assembly1_B	Mapping the E2 Glycoprotein of Alphaviruses	*Sindbis virus*	10.8	0.665
**L segment**				
**MKWV N-terminus endonuclease, 182 a.a.**				
6qvv assembly1_A	Structure and function of phenuiviridae cap snatching endonucleases	*Toscana virus*	36.3	0.854
6qw0 assembly1_A	Structure and function of toscana virus cap snatching endonucleases	*Toscana virus*	35.2	0.836
6qvv-assembly2_B	Structure and function of phenuiviridae cap snatching endonucleases	*Toscana virus*	36.3	0.822
6ntv-assembly3_C	SFTSV L endonuclease domain	*Severe fever with thrombocytopenia syndrome virus*	32.6	0.819
**RdRp, 697 a.a.**				
7eei-assembly1_A	Structure of Rift Valley fever virus RNA-dependent RNA polymerase	*Rift Valley fever virus*	57.6	0.728
6y6k-assembly1_A	Cryo-EM structure of a Phenuiviridae L protein	SFTS virus AH12	40.4	0.722
8asg-assembly1_A	Structure of the SFTSV L protein bound in a resting state [RESTING]	SFTS virus AH12	40.7	0.697
8as7-assembly1_A	Structure of the SFTSV L protein stalled at early elongation [EARLY-ELONGATION]	SFTS virus AH12	41.1	0.695
**RNA-dependent RNA polymerase complex, 2098 a.a.**				
8as6-assembly1_A	Structure of the SFTSV L protein bound to 5' cRNA hook [5' HOOK]	SFTS virus AH12	32.4	0.832
8asg-assembly1_A	Structure of the SFTSV L protein bound in a resting state [RESTING]	SFTS virus AH12	32	0.828
7alp-assembly1_U	Severe fever with thrombocytopenia syndrome virus (Phenuiviridae) L protein	Phlebovirus WCH/97/HN/China /2011	25.3	0.821

6l42-assembly1_A	Structure of severe fever with thrombocytopenia syndrome virus L protein	Phlebovirus WCH/97/HN/China /2011	24.8	0.808
7eei-assembly1_A	Structure of Rift Valley fever virus RNA-dependent RNA polymerase	Rift Valley fever virus	34	0.794
6y6k-assembly1_A	Cryo-EM structure of a Phenuiviridae L protein	SFTS virus AH12	27.3	0.763

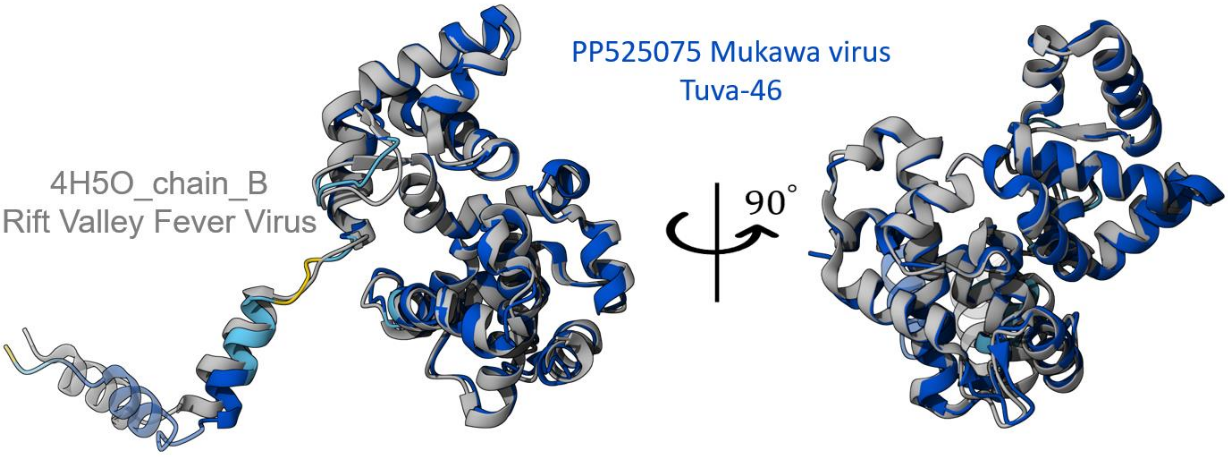

Figure S1. Superposition of the MKWV Tuva-46 nucleoprotein tertiary structure model (pLDDT color) and crystal structure of Rift Valley fever virus nucleoprotein (PDB ID: 4H5O, gray).

Table S5. Identity level of nucleotide and amino acid sequences (%) of identified Russian MKWV isolates with a MKWV prototype isolates (Excel document).

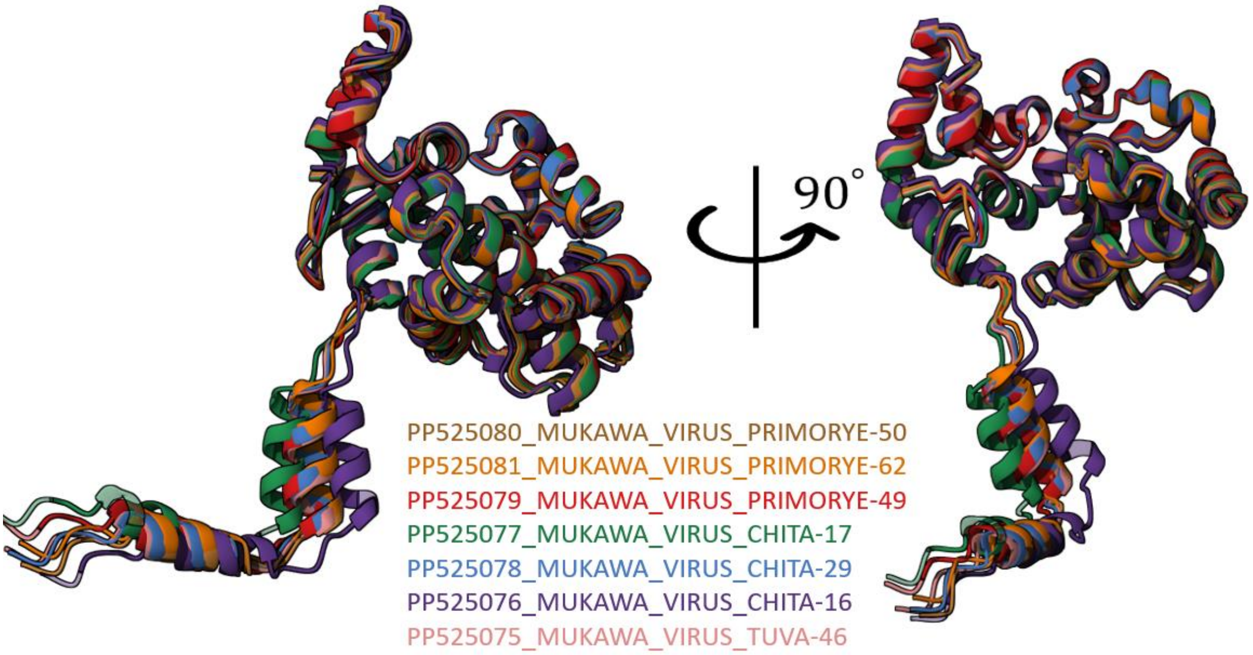

Figure S2. Superposition of the MKWV nucleoprotein tertiary structure models from Russian isolates.
