# Supplementary figures and images for: Expanding diversity of tick-borne phleboviruses (Phlebovirus mukawaense, Mudanjiang phlebovirus, Gomselga Virus, and Onega tick phlebovirus) in Russia
Source: PLoS One. 2026 Jun 2;21(6):e0349564. doi: 10.1371/journal.pone.0349564 (PMC13229322; doi:10.1371/journal.pone.0349564)

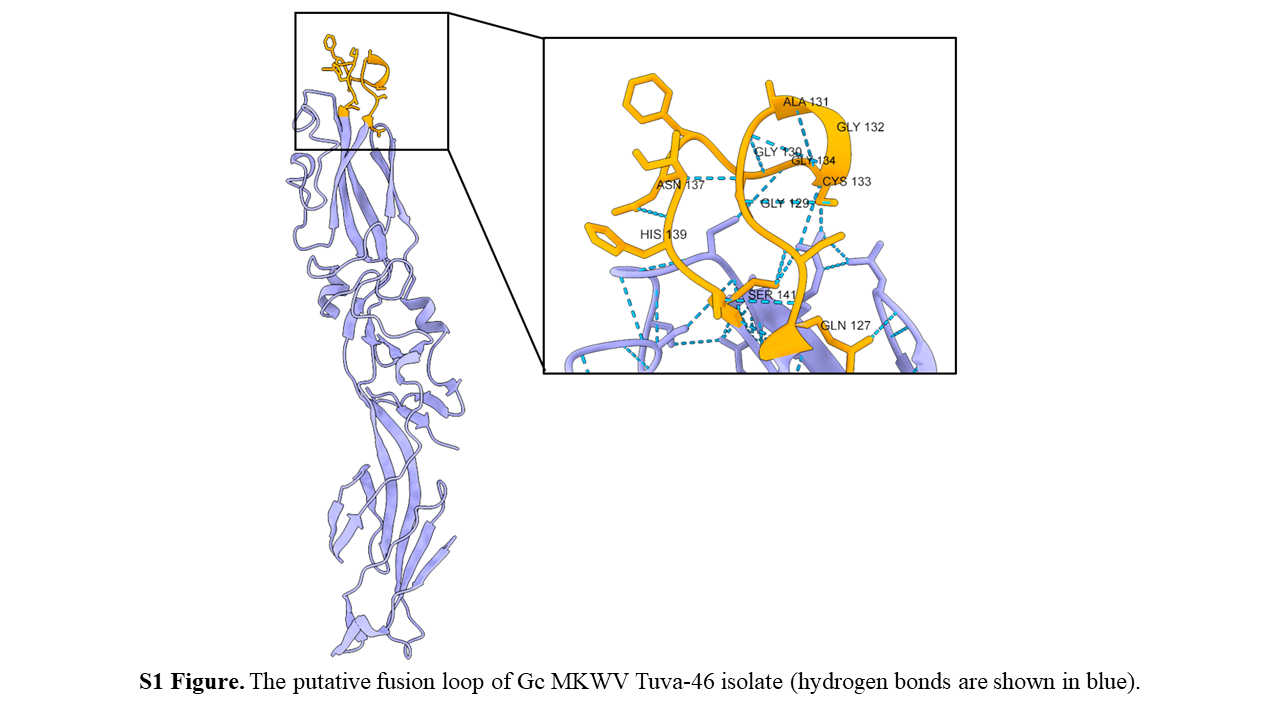

Supplement: S1 Fig — (TIF) [file pone.0349564.s004.tif]

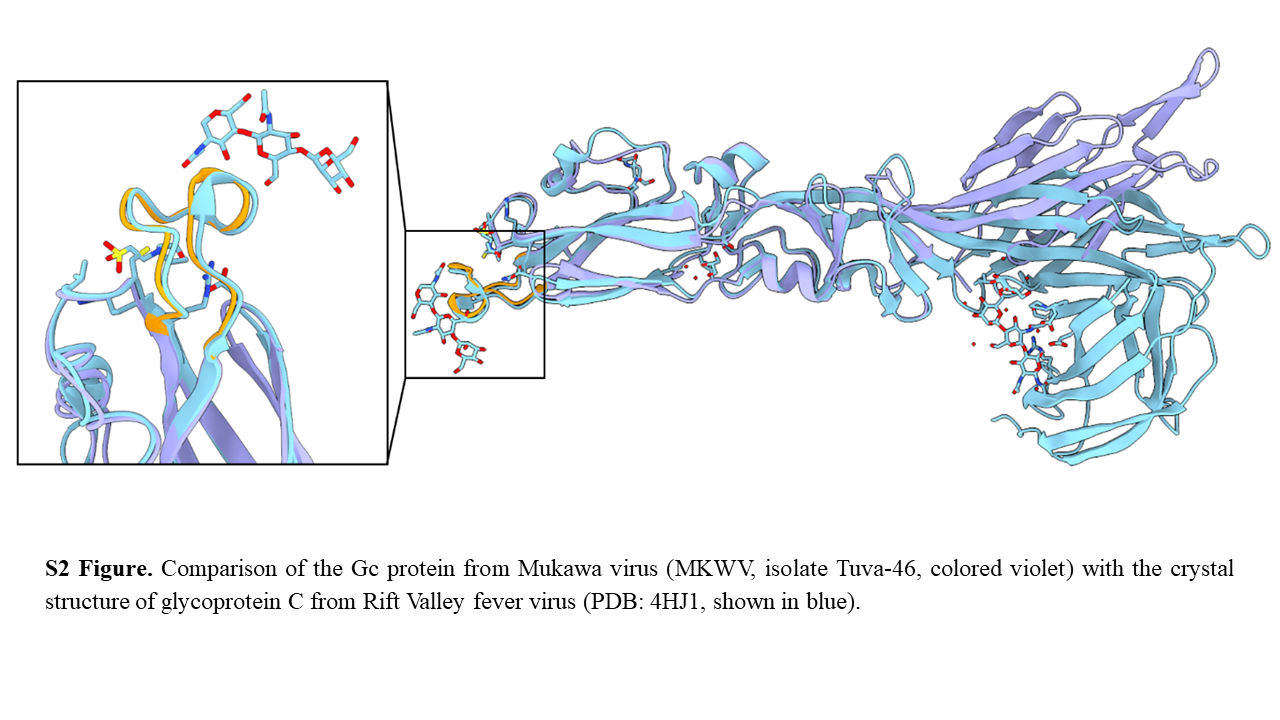

Supplement: S2 Fig — (TIF) [file pone.0349564.s005.tif]

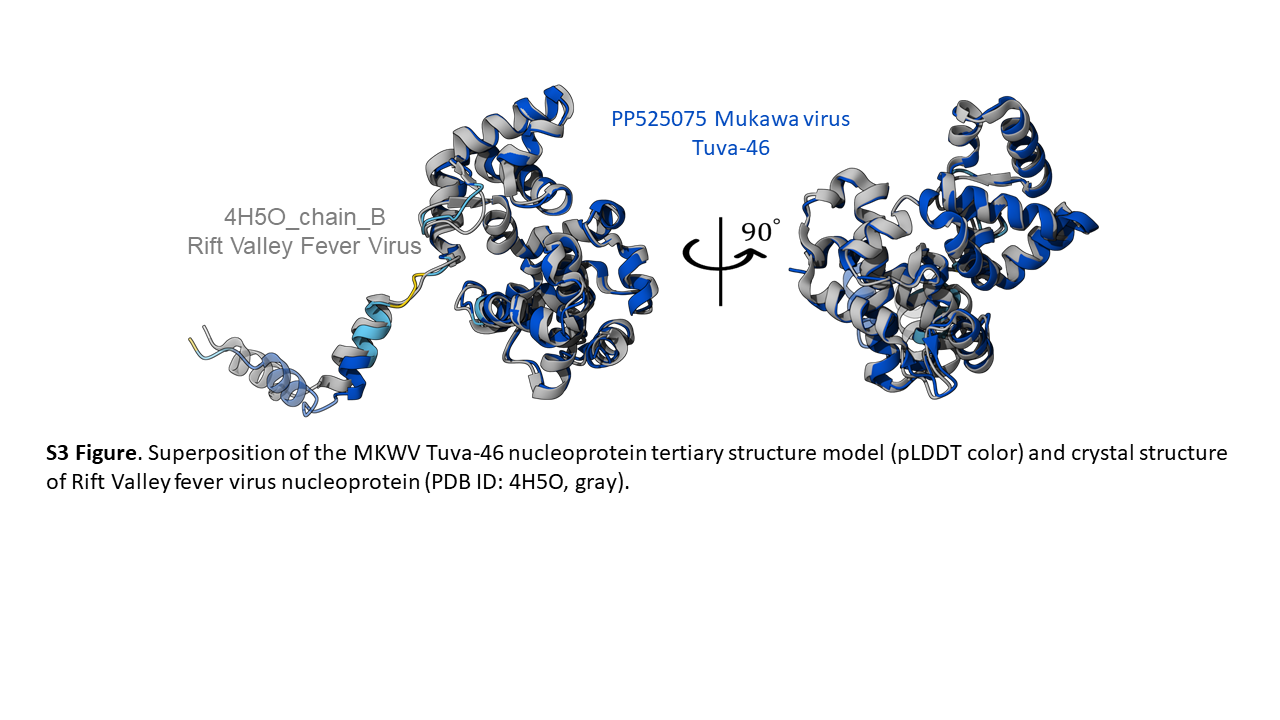

Supplement: S3 Fig — (TIF) [file pone.0349564.s006.tif]

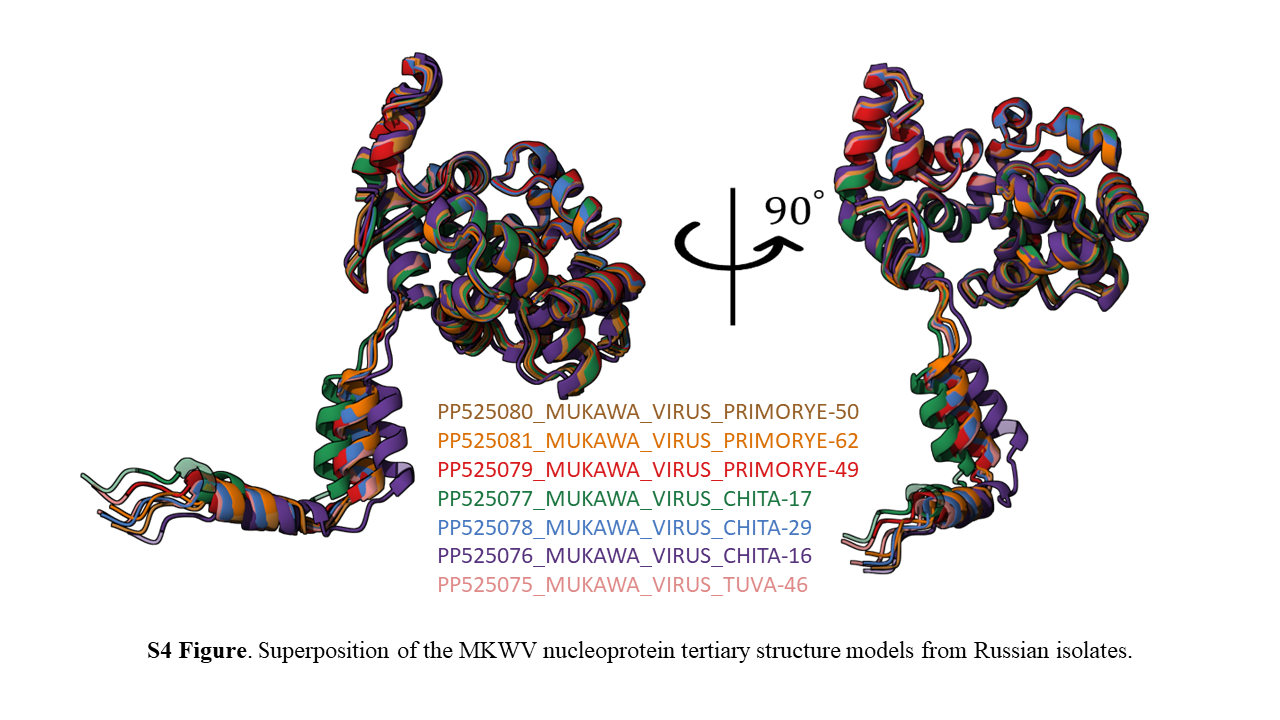

Supplement: S4 Fig — (TIF) [file pone.0349564.s008.tif]
